# Reproductive, maternal, neonatal and child health in conflict: a case study on Syria using Countdown indicators

**DOI:** 10.1136/bmjgh-2017-000302

**Published:** 2017-09-14

**Authors:** Jocelyn DeJong, Hala Ghattas, Hyam Bashour, Rima Mourtada, Chaza Akik, Amelia Reese-Masterson

**Affiliations:** 1 Epidemiology and Population Health Department, Faculty of Health Sciences, American University of Beirut, Beirut, Lebanon; 2 Faculty of Health Sciences, Center for Research on Population and Health, American University of Beirut, Beirut, Lebanon; 3 Department of Family and Community Medicine, Damascus University, Damascus, Syrian Arab Republic; 4 Research Adviser in Nutrition, Food Security and Livelihoods, International Medical Corps, Beirut, Lebanon

**Keywords:** public health, maternal health, child health

## Abstract

**Introduction:**

Women and children account for a disproportionate morbidity burden among conflict-affected populations, and yet they are not included in global accountability frameworks for women’s and children’s health. We use Countdown to 2015 (Millennium Development Goals) health indicators to provide an up-to-date review and analysis of the best available data on Syrian refugees in Jordan, Lebanon and Turkey and internally displaced within Syria and explore data challenges in this conflict setting.

**Methods:**

We searched Medline, PubMed, Scopus, Popline and Index Medicus for WHO Eastern Mediterranean Region Office and relevant development/humanitarian databases in all languages from January 2011 until December 2015. We met in person or emailed relevant key stakeholders in Lebanon, Jordan, Syria and Turkey to obtain any unpublished or missing data. We convened a meeting of experts working with these populations to discuss the results.

**Results:**

The following trends were found based on available data for these populations as compared with preconflict Syria. Birth registration in Syria and in host neighbouring countries decreased and was very low in Lebanon. In Syria, the infant mortality rate and under-five mortality rate increased, and coverage of antenatal care (one visit with a skilled attendant), skilled birth attendance and vaccination (except for DTP3 vaccine) declined. The number of Syrian refugee women attending more than four antenatal care visits was low in Lebanon and in non-camp settings in Jordan. Few data were available on these indicators among the internally displaced. In conflict settings such as that of Syria, coverage rates of interventions are often unknown or difficult to ascertain because of measurement challenges in accessing conflict-affected populations or to the inability to determine relevant denominators in this dynamic setting.

**Conclusion:**

Research, monitoring and evaluation in humanitarian settings could better inform public health interventions if findings were more widely shared, methodologies were more explicit and globally agreed definitions and indicators were used consistently.

**Summary box**

**What is already known about this topic?**

- The health of women and children is disproportionately affected in conflict situations being heavily dependent on a functioning health system and vulnerable to economic and societal disruption induced by conflict.
- Reaching conflict-affected populations was the unfinished agenda of the Millennium Development Goals and should be a priority of the sustainable development goals.

**What are the new findings?**

- According to available data, coverage rates of most key evidence-based interventions in reproductive, maternal, newborn and child health declined in Syria; among refugees in neighbouring countries the picture was more mixed as compared with preconflict Syria.
- In conflict settings such as that of Syria, coverage rates of such interventions are often unknown or difficult to ascertain.

**Recommendations for policy**

- Much research is being done on those forcibly displaced from the Syrian conflict, but better and more accessible and timely data, more use of standardised definitions and more explicit methodological approaches would strengthen the evidence-base for intervention to improve their health.
- Special attention to data constraints on the health situation of women and children in conflict settings – where both coverage and data availability are often undermined - is needed in global health accountability frameworks.

## Introduction

Women and children account for a disproportionate morbidity burden among conflict-affected populations,^1^ being heavily dependent on a functioning and responsive health system and vulnerable to economic and societal disruption induced by conflict. Yet conflict has not been, until now, sufficiently included in global accountability frameworks for the health of women and children.^2^ Addressing maternal health in conflict settings is in many ways the unfinished agenda of the Millennium Development Goals (MDGs). The Sustainable Development Goals (SDGs) note the need to focus on conflict-affected populations but do not provide specific goals in this regard.^3 4^

The Countdown to 2015 initiative for Reproductive, Maternal, Newborn and Child Health (RMNCH)^5 6^ focused on the 75 countries where more than 95% of all maternal and child deaths occur and tracked coverage, aimed to stimulate coverage assessment mechanisms and support country progress towards achieving MDG4 and MDG5. Countdown is now shifting emphasis to the SDGs and a new set of indicators is being agreed on to 2030.^7^

Countdown to 2015^6^ did not deliberately select countries in conflict, yet of the 10 countries with the highest under-five mortality rates worldwide, eight are conflict affected.^8^ Globally, the nature of conflict is changing, becoming increasingly intrastate, with greater civilian toll and a shift from low-income to middle-income settings.^9^ These changing parameters of conflict make reaching those most in need through evidence-based public health interventions particularly challenging.

Within the Arab region, Countdown to 2015 included five countries—Egypt, Iraq, Morocco, Sudan and Yemen—four of which have experienced political instability or conflict during the past decade. Syria is not included, given the fact that, by 2008, it had achieved 85% of its MDG4 target and 68% of its MDG5 target.^10^ Syria’s maternal mortality ratio fell from 107/100 000 in 1993 to 56 in 2008.^10^ Its infant mortality rate similarly dropped from 34.6 deaths per 1000 live births to 18 in the same period.^10^ Just before the conflict began, skilled attendance at delivery was at 96%.^11^ Nevertheless, inequalities across regions were evident preconflict.^10 12^

The current crisis in Syria has led to a widespread deterioration in what was a well-functioning and predominantly public healthcare system, with the destruction or disruption of health facilities, and exodus of health professionals. Additionally, population movement and the corrosion of public services and infrastructure^13 14^ have increased the risk of outbreaks of previously controlled epidemics.^14^ More than half the population now lives in poverty, with 7.9 million people becoming poor since the beginning of the crisis.^15^ These structural and economic realities reduce women’s access to reproductive health (RH) services.^16^

**Figure 1:**
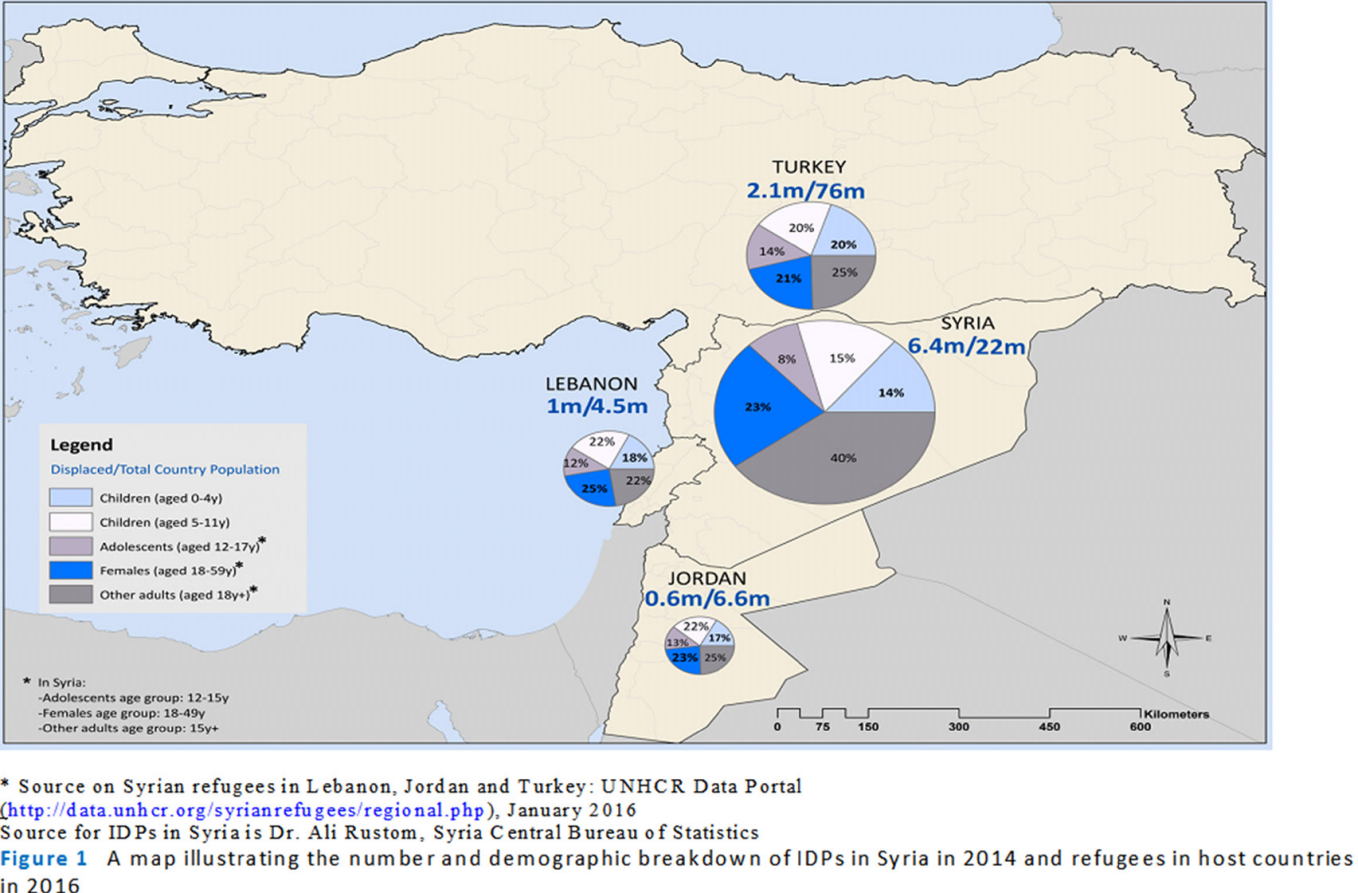
A map illustrating the number and demographic breakdown of IDPs in Syria in 2014 and refugees in host countries in 2016.

Stark demographic shifts have also occurred, with 6.5 million Syrians internally displaced and 3.7 million living as refugees in neighbouring Turkey, Lebanon and Jordan (2016).^17^ Figure 1 shows current figures for internally displaced populations (IDPs) in 2014, and refugees in 2016 as a ratio over total country populations for countries hosting displaced Syrians, as well as their age and gender breakdowns. In total, women and children under the age of 18 account for 60% of IDPs and 76% of refugees in neighbouring countries, indicating a heavy burden on health and educational services in both Syria and host countries. Table S1 details the rights and status of Syrian refugees in host countries. Within Syria, public health responsibility is fragmented between opposing forces, which run parallel health systems with shifting geographic areas of coverage, making data collection particularly challenging.

10.1136/bmjgh-2017-000302.supp1 Supplementary file 1

In this paper, we use the Countdown to 2015^6^ framework and set of RMNCH coverage and health status indicators to provide an up-to-date review and critique of the best available data focusing on the conflict-affected Syrian population both within Syria (including non-displaced and IDPs) and those living as refugees in neighbouring countries.^18 19^ We highlight potential challenges in so doing and insights that can be gained by using existing internationally endorsed monitoring frameworks to systematically analyse how conflict affects measures of health status and coverage of evidence-based interventions. In so doing, we aim to draw lessons of relevance for other conflict situations, particularly as the SDGs begin to be implemented.

## Methods

### Published literature

We searched Medline, PubMed, Scopus, Popline and Index Medicus for WHO EMR (WHO Eastern Mediterranean Region) databases for publications from January 2011 until December 2015. We used Medical Subject Heading Terms and keyword search strategies with various combinations of terms related to the relevant Countdown indicators, with the additional descriptor of Syria and Syrian refugees. A search strategy was developed for Medline and adapted for the other databases (online supplementary appendix 1). We omitted malaria and AIDS indicators as the former is not endemic in the study countries and the latter has very low prevalence.^10^ We further searched Google Scholar and conducted purposive searching of specific relevant journals and hand searching of bibliographies of included articles. We developed inclusion and exclusion criteria to assess the published literature. We included original research studies that reported on Countdown indicators or proxy determinants on preconflict Syria, and during conflict among the Syrian population resident in Syria, the internally displaced or refugees in the neighbouring host countries (Jordan, Lebanon and Turkey). Only peer-reviewed articles with primary data or secondary data analysis of relevant indicators were included. We extracted the following descriptive information from included sources: nationality, residence, institution of the first author; main data sources, year of main data source, study population (Syrian residents, internally displaced or refugees); sample size and characteristics; language; proxy determinants or direct indicators; research design and whether it was peer reviewed or not.

All retrieved articles were imported into EndNote reference manager, and duplicates were deleted. Title and abstract screening followed by full text screening were conducted by two researchers, and any disagreements were discussed with a third researcher to reach consensus.

### Grey literature

We searched databases relevant to the humanitarian efforts concerning the Syrian conflict and regional development: Relief Web, UNHCR Syria Regional Refugee Response Portal, United Nations Development Programme and WHO Eastern Mediterranean Regional Office websites for the same time period.

The initial search retrieved thousands of documents of various types (such as situation reports, funding appeals, infographics and meeting minutes), which reported time periods extending from 1 week to 1 year. Consequently, the study team examined the content of each type of report and categorised them to assess comprehensiveness and relevance. Because data on the humanitarian crisis is constantly updated, we opted to include only data from quarterly, midyear and annual reports. Quality appraisal took into account whether the publication was data driven (based on original or secondary data) or not, and whether there was a specified study methodology. Because of inconsistencies and incompleteness in data reported in published reports, we contacted governmental and non-governmental entities to fill these gaps. Key informants and governmental agencies were not always responsive or ready to share data on certain indicators even when data were available. The tables on results therefore differentiate between data that were not available (N/A) (meaning they do not exist) and not obtainable (N/O) (meaning they exist but we could not obtain them).

### Additional strategies

Given the paucity in English published and grey literature on outcomes of interest among Syrian refugees in Turkey in particular, a team member travelled to Istanbul and Ankara to meet with representatives of key non-governmental organisations and academics.

An expert meeting was conducted in Beirut, Lebanon, in January 2016 with stakeholders working with IDPs or Syrian refugees from countries of interest (Syria, Lebanon, Jordan and Turkey) to review and discuss the results and to obtain any missing relevant data. The cut-off date for updating the statistics used in the paper was 15 March 2016.

### Literature analysis and synthesis

We extracted all data on Countdown indicators from identified literature based on the following geotemporal categories: Syria preconflict, Syria since 2011 and Syrian refugees in Lebanon, Jordan, and Turkey. Data were tabulated using the Countdown to 2015^6^ framework of indicators.

Additionally, we adapted an organisational framework^20 21^ to portray a model for how the Syrian conflict and responses to it have interacted with relevant health status and coverage indicators since the start of the conflict.

### Role of funding source

The funder played no role in the collection, analysis or publication of data.

## Results

**Figure 2:**
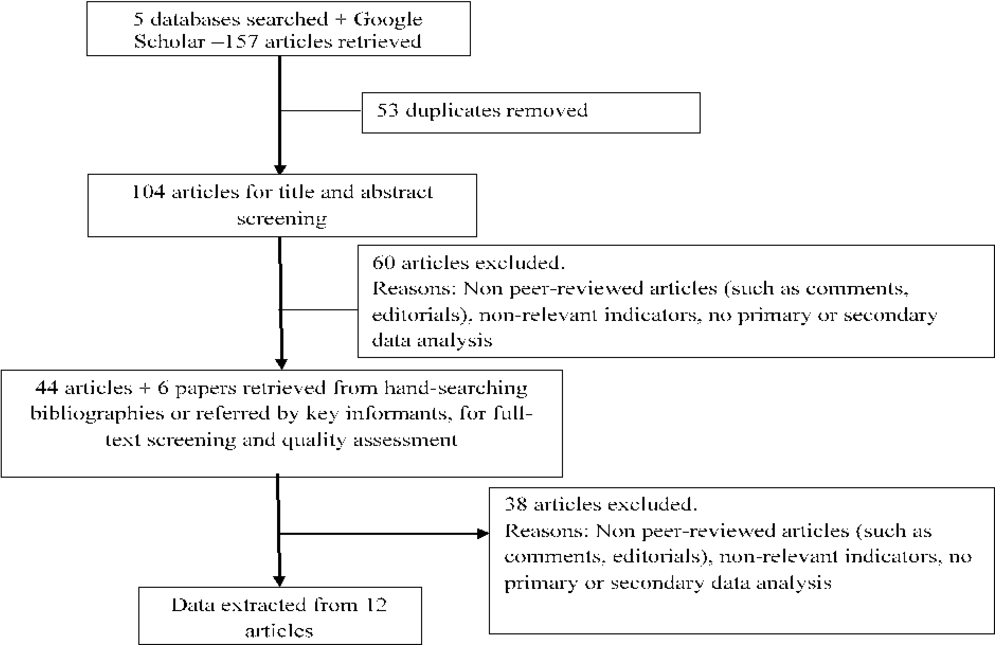
Flow diagram for review of published literature.

We retrieved 157 published articles (figure 2), two-thirds of which were editorials or correspondence articles referencing data from grey literature and were therefore excluded. Only 12 articles were based on primary or secondary data analysis of relevant indicators. Among these 12, only four articles had their first author from a host country of refugees and based in a local institution (one from Jordan, one from Lebanon and two from Turkey), and only four articles sought ethical approval while two reported that it was not required. Three studies targeted Syrian refugees in Jordan, five in Lebanon and three in Turkey, and one focused on health professionals working with refugees in Jordan; none were on the internally displaced. Over 3000 documents produced by over 150 agencies were identified through the grey literature search and screened for data extraction.

### Organisational framework

**Figure 3:**
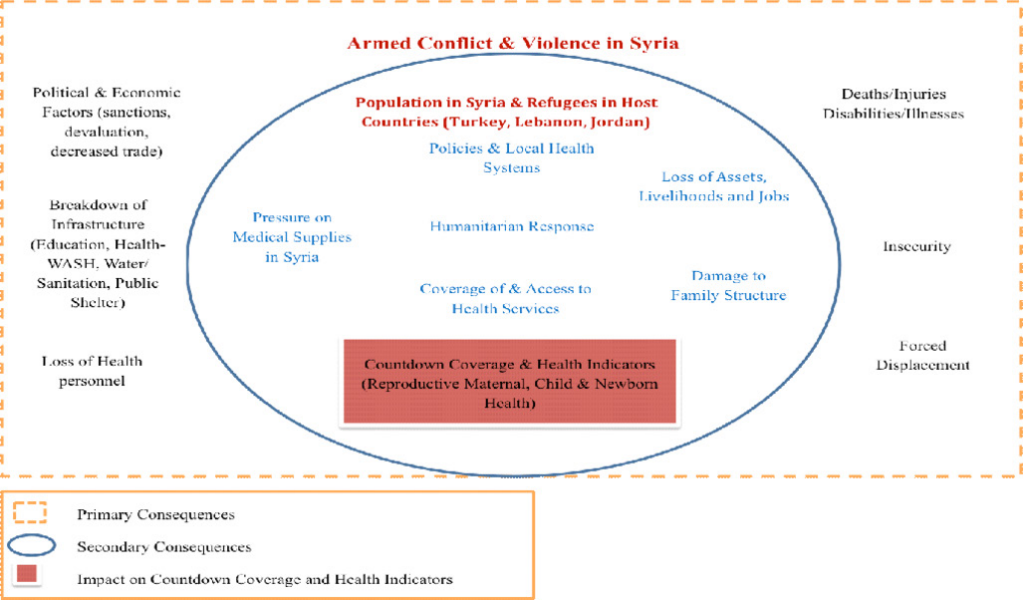
An organisational frame work describing the direct consequences of armed conflict and violence in Syria.

Figure 3 presents an organisational framework based on the literature and identifies various pathways^20–22^ through which the armed conflict may have influenced public health^23 24^ and RMNCH indicators^25 26^ among Syrians in Syria and in host countries.

#### Countdown indicators

Findings from the review of published and available grey literature on the Syrian conflict relevant to RMNCH Countdown to 2015 indicators are presented for the populations in the three host countries and Syria.

#### Demographics

Table 1 outlines numbers for the under 5-year-old population as well as birth, mortality and fertility rates.

**Table 1:** Demographic indicators in Syria preconflict and in conflict and of Syrian refugees in neighbouring countries

	Syria preconflict	Syria in conflict	Lebanon	Jordan (non-camp)	Jordan (camps)	Turkey
Total children U5 population	2 683 000 (Personal communication with Ali Rustom, Syrian Central Bureau of Statistics, 2015)	2 877 000 (Personal communication with Ali Rustom, Syrian Central Bureau of Statistics, 2015)	190 494^17^*	Total 108 956^17^* Zaatari 15, 692^17^* Azraq 6 278^17^*		458,380^17^*
Total births (per year)	548 000^59^†	460 000 (Personal communication with Ali Rustom, Syrian Central Bureau of Statistics, 2015)	43 000 (Personal communication with Ms Randa Hamadeh, MoPH, Lebanon, 2015)	N/O	Zaatari 3730^34^* Azraq 661^60^^*^	20 000* (on average)^30^†
Birth registration (%)	90.9^11^‡	60 (Personal communication with Ali Rustom, Syrian Central Bureau of Statistics, 2015)^e^	23.0^28^†	N/A	N/A	N/O
Total deaths for children U5	8000^61^^*^	N/O	N/O	N/O	Zaatari 50.0^34^* Azraq 5.0^62^^*^	N/O
U5 mortality rate (per 1000 live births)	21.4^11^‡	25.1^22^*	N/O	N/A	Zaatari 25.2^34^* Azraq 15.3^62^^*^	N/O
Neonatal deaths as a percent of U5 deaths	50.0^11^‡	N/A	N/O	N/A	Zaatari 56.0^34^* Azraq 100.0^62^*	N/A
Neonatal mortality rate (per 1000 live births)	12.9^11^‡	7 (4.5–10.4)^63^*§	N/O	N/A	Zaatari 14.1^34^* Azraq 15.3^64^*	N/A
Infant mortality rate (per 1000 live births)	17.9^11^‡	23.3^22^*	N/O	N/A	Zaatari 1.5^65^* N/O	
Stillbirth rate (per 1000 total births)	12.4^66^*	N/A	N/A	N/A	Zaatari 14.2^33^* Azraq 6.1^62^*	N/O
Maternal mortality ratio	52^11^‡	62.7^22^*	N/A	N/A	Zaatari 1 out of 81,042^67^*	N/A
Total fertility rate (sum of age-specific birth rates for every 5-year-old age group * 5)	3.5^11^‡	3^68^*, §	N/A	N/O	N/O	N/O
Adolescent birth rate (per 1000 adolescent women)	60^69^*	40^70^*, §	N/A	N/A	N/A	N/O

The following indicators were not included due to the lack of data: total maternal deaths, lifetime risk of maternal deaths, preterm birth rate and causes of U5 mortality.

*Surveillance/monitoring data.

†One-time thematic report.

‡Population-based surveys.

§Estimates (lower limit–upper limit).

¶Peer-reviewed article.

N/A, not available (ie, data do not exist to our knowledge); N/O, not obtainable (ie, data exist but authors unable to obtain).

### Births

In Syria and host countries, births to Syrian nationals often go unregistered due to security threats, missing identity documents and other legal or logistical barriers.^27^ This can have serious consequences, including increased risk of exposure to violence, abuse or exploitation; risk of statelessness; difficulty accessing healthcare and education; difficulty in voluntary return, crossing borders and proving Syrian citizenship on return; and difficulty obtaining work or legal services later in life.^28^

Birth registration among refugees in Lebanon is very low. In Jordan and Turkey, there are no representative numbers on birth registration among Syrian refugees. In Zaatari camp (Jordan), over 1400 children born between November 2012 and July 2013 did not receive birth certificates.^28^ The Turkish Prime Ministry Disaster and Emergency Management Authority (AFAD) registered about 30,000 Syrian births between 2011 and 2014^29^ (out of some 60 000 births since the start of the crisis).^30^

### Birth outcomes

Data on preterm birth rate and stillbirth are lacking for all settings. Reports indicate that 26% of Syrian refugee births in Lebanon were preterm,^31^60% of neonatal deaths in Zaatari refugee camp in Jordan were due to prematurity^32^ and 9.4 (per 1000 total births) were stillbirths in Zaatari (down from 14.2 the previous year).^33 34^

Limited data were available on adolescent birth rates; 8.5% of deliveries in Zaatari camp were to adolescent girls in 2014.

### Deaths

Table 1 outlines available data on maternal, neonatal, infant and child mortality. Given the difficulty of obtaining accurate data on maternal and child deaths due to the conflict, these figures are not disaggregated to include conflict-related mortality. Estimates indicate a reduction in neonatal mortality rate in Syria, possibly due to geographical exclusion of hard-to-reach areas. Part of the excess mortality burden due to conflict is occurring in under 5 year olds, which is partially due to reduced healthcare access in conflict-affected areas.^35^

### Maternal and newborn health

#### Demand for family planning

In Syria in 2009, 83.6% of demand for family planning was met (table 2), and this is estimated to have increased within Syria to 88.7%^11 36^ and among non-camp refugees living in Amman, Jordan. These numbers are lower in Lebanon (63.9%) (table 2), where access to RH services is reportedly hindered by cost, travel and limited female healthcare providers.^31^

**Table 2:** Indicators of maternal and newborn health in Syria preconflict and in conflict and in Syrian refugees in neighbouring countries

	Syria preconflict	Syria in conflict	Lebanon	Jordan non-camp	Jordan camps	Tuñrkey
Need for family planning satisfied	83.6^11^ ^a^	88.7 36 ^f^*	63.9^31^*	85.6^71^*^c^	N/A	N/A
ANC (at least one visit with a skilled attendant)	87.7^11^ ^a^	62.0^22^ ^c^	87.0^53^ ^a^	88.7^54^ ^a^	N/A	N/A
ANC (at least four visits with any attendant)	63.7^11^ ^a^	N/A	49.3^53^ ^a^	15.6% (3–4 visits)^54^ ^a^ 38.2% (>4 visits)^54^*	Zaatari 71.0^72^ ^b^ Azraq 86.0^62^ ^b^	N/A
Neonatal tetanus protection (%)	37.8^69^*	N/A	N/A	N/A	N/O	N/A
Delivery care/ Skilled attendant at birth	96.2^11^ ^a^*	72.0^22^ ^c^	99.4^53^ ^a^*	N/A	Zaatari 100.0^72^ ^b^ Azraq 100.0^64^ ^b^	96.0 in camps^41^ ^a^ 97.0 outside of camps^41^ ^a^
Caesarean section (%)	26.4^11^ ^a^	46.0 (Personal communication with Bashar Kourdi, 2015)^73^ ^e*^	35.3^40^ ^d^	26.9^54^ ^a^	Zaatari 28.0^72^ ^b^ Azraq 19.0^62^ ^b^	N/A
Postnatal care (%)	27.2^11^ ^a^	N/A	8.3^74^ ^c^	N/A	Zaatari 47.0 †^72^ ^b^ Azraq 52.0^64^ ^b^	N/A

a Population-based surveys.

b Surveillance/monitoring data.

c One-time thematic report.

d Peer-reviewed article.

e Estimates (lower limit–upper limit).

*Estimates were calculated using the contraceptive prevalence rate conversion using Countdown Technical Notes (^5^).

†At least three visits within 6 weeks of delivery.

‡Data from the largest maternity hospital in Damascus.

ANC, antenatal care; N/A, not available (ie, data do not exist to our knowledge); N/O, not obtainable (ie, data exist but authors unable to obtain).

There were no reliable figures on fertility among refugees or within Syria. This issue of fertility and forced displacement internationally is a subject of debate, with some attributing a possible increase in fertility to the need to replace lost children, whereas others suggest that fertility is decreasing because of the stress and uncertainties of displacement.^37–39^

### Antenatal care (ANC)

Within Syria, coverage of at least one ANC visit with a skilled professional has declined from 87.7% to 62%, likely related to the widespread displacement, the exodus of healthcare professionals and the destruction of health facilities. Coverage in Lebanon and Jordan remains at similar levels to prewar Syria, whereas coverage of at least four ANC visits is much lower (in non-camp refugee populations) (table 2). No data on ANC were available for Turkey.

### Skilled attendant at delivery

Rates of skilled birth attendance at delivery have dropped in Syria from 96.2% to 72%, whereas they remain almost universal among refugees in neighbouring countries, likely as a result of special provisions by UNHCR in Lebanon and Jordan (table 2).

### Caesarean section

Available data indicate increases in C-section rates both within Syria (where nationally the rate was 26.4% preconflict) and among refugees since the conflict began (table 2).^11^ Data from the largest public maternity hospital in Damascus show C-section rates increasing from 29% in 2010 to 46% in 2014 (Personal communication with Bashar Kurdi, 2015). This could be explained by the need for women and their physicians to schedule delivery around the security situation. It could however also be an unintended consequence of interventions introduced to help women reach RH services, such as the voucher system introduced to enable women to obtain free maternal health and emergency obstetric services including C-section.

In Lebanon, in 2013, 35.3% of Syrian refugee deliveries in UNHCR-contracted hospitals were C-sections,^40^ and the corresponding figures were 36% in 2014 and 33.7% in 2015 out of all referrals for delivery (Personal communication with Marie Akiki and Dr Diana Aoun, UNHCR, 2016). Potential explanations for this high rate are limited access to and utilisation of ANC increasing the risk for high-risk pregnancies, privatised healthcare system, predominance of male obstetric providers or UNHCR’s financial coverage of most C-sections.^40^

### Postnatal care

Postnatal visits to health facilities were low at 27% in Syria in 2009 (table 2). Limited data are available for an analysis of changes since the crisis; however, within a camp setting in Jordan, where several specialised RH facilities have been established, postnatal care is higher at approximately 50%. No data on postnatal care coverage for the newborn were identified.

### Child health

Table 3 shows that available data on coverage of measles, diphtheria-tetanus-pertussis (DTP3) and *Haemophilus influenzae* type B (Hib) vaccinations in Syria was high prior to the conflict (at 81.9%, 99% and 82.1%, respectively) and has suffered setbacks during the war except for DTP3 vaccine coverage, which increased. Refugee children are also reported to have lower than optimal coverage rates with scarce data showing large variations in coverage.^41–43^ Other indicators of child health were also high prior to the conflict, but no data on care seeking and antibiotic treatment for pneumonia nor for oral rehydration therapy for diarrhoea in Syria or neighbouring countries exist for the period since the conflict began (table 3). This highlights the partial development and implementation of health policies concerning these interventions in this context and the lack of surveillance data.

**Table 3:** Indicators of child health in Syria preconflict and in conflict and in Syrian refugees in neighbouring countries

	Syria preconflict	Syria in conflict	Lebanon	Jordan non-camp	Jordan camps	Turkey
Measles immunisation coverage (%) for 12–23 months	81.9^11^*	75.5^75^*	59.4^53^*	31.2^42^* of children U5 86.6^43^* of children U5	76.9 in Za'atari camp^42^* of children U5	58.7%–71.8% (out of camps vs in camps^41^* Of children <10 years old
Three doses of combined DTP3 vaccine (%)	82.1^11^*	91.8^75^*	N/A	N/A	N/A	N/A
Three doses of combined Hib vaccine (%)	99.0^76^ ^b^	91.8^75^*	N/A	N/A	N/A	N/A
Care seeking for pneumonia for children U5 (%)	85.7^11^*	N/A	N/A	N/A	N/A	N/A
Antibiotic treatment of pneumonia for children U5 (%)	63.9^11^*	N/A	N/A	N/A	N/A	N/A
Oral rehydration therapy and continued feeding (%)	84.4^11^*	N/A	N/A	N/A	N/A	N/A

*Population-based surveys.

N/A, not available (ie, data do not exist to our knowledge); N/O, not obtainable (ie, data exist but authors unable to obtain).

### Nutrition

#### Underweight, wasting, stunting prevalence

The nutritional status of under-5-year-old children in preconflict Syria reflected medium levels of underweight, wasting and stunting (table 4); these have remained relatively constant. It is probable that these data do not cover hard-to-reach and besieged areas and are underestimates of the true magnitude of malnutrition. In contrast, surveys of Syrian refugees in Lebanon and Jordan show much lower rates of undernutrition (table 4). Where data exist on differentials between camp and non-camp populations in Jordan, camp-dwelling children were shown to have higher rates of undernutrition, although lower than those inside Syria. Improved indicators in refugee settings may reflect the large investments made in capacity building for the management of malnutrition, as well as the implementation of screening and treatment protocols.^44^

**Table 4:** Nutritional profile of Syrian children, preconflict, in conflict and as refugees in neighbouring countries

	Syria preconflict	Syria in conflict	Lebanon	Jordan non-camp	Jordan camps	Turkey
Underweight prevalence (%)	10.3^11^*	13.8^77^*	2.6^78^*	2.7 79*	Zaatari 4.0^79^*	N/A
Stunting prevalence (%)	23.0^11^*	22.3^77^*	18.6^78^*	9.0^79^*	Zaatari 17.0^79^*	N/A
Wasting prevalence (%)	9.3^11^*	7.2^77^*	2.2^78^*	0.8^79^*	Zaatari 1.2^79^*	N/A
Exclusive breastfeeding (%)	42.6^11^*	59.1^77^*	25.0^78^*	36.0^79^*	Zaatari 46.4^79^*	N/A
Early initiation of breastfeeding (%)	45.5^11^*	N/A	31.3^78^*	48.7^79^*	Zaatari 57.0^79^ a	N/A
Low birth weight incidence (%)	10.3^11^*	N/A	N/A	N/A	Zaatari 2.0 †^72^‡ Azraq 4.0 †^64^§	N/A
Vitamin A supplementation (%)	34.8^11^*	75.5^75^*	52.3^53^*	4.5^42^*	32.8^42^*	N/A

*Population-based surveys.

†Indicated as underreported by authors of cited article.

‡Surveillance/monitoring data.

§One-time thematic report.

#### Infant and young child feeding (IYCF)

Early initiation of breastfeeding in preconflict Syria was estimated at around 45% (table 4) and is lower in refugees in Lebanon and higher in Jordan. Parallel trends in breastfeeding exclusivity in under 6 month olds were observed (lower rates in Lebanon and non-camp settings in Jordan, and higher rates in Zaatari camp). Reduced breastfeeding in refugees may result from maternal stress related to displacement,^20^ possibly mitigated by the implementation of IYCF programmes^45 46^ in Zaatari. Interestingly, exclusive breastfeeding is reported to be higher in Syria in conflict at 59.1%^44^ than preconflict at 42.6%^11^ (table 4), possibly due to financial constraints or disruption of infant formula supplies and markets. Complementary feeding data are lacking.

#### Vitamin A supplementation

A recent meta-analysis has shown that vitamin A supplementation (VAS) results in child mortality reductions of 11%.^47^ About one-third of 6-month-old to 59-month-old Syrian children received at least one vitamin A dose preconflict, and VAS programs have been implemented for Syrian refugees in host countries, achieving over 50% coverage in Lebanon, 33% in Zaatari and under 5% in children living outside of camps in Jordan (table 4). Although numbers for children who received VAS are unavailable for Syria in conflict, UNICEF was able to dispense 34% of its target of VAS, administered with the measles, mumps and rubella vaccination.^48^

#### Policies

Table S2 summarises the status of Countdown policy indicators in Syria preconflict and postconflict and in host countries postconflict.

#### Financing

Table S3 summarises Countdown financing indicators in Syria preconflict and during conflict, as well as in host countries.

## Discussion

In this paper, we have used the Countdown to 2015^6^ set of RMNCH indicators applied to the case of populations affected by the Syrian conflict and summarised available (and obtainable) data on coverage of evidence-based interventions across the continuum of care. Demographic data show a decline in birth registration among Syrians in Syria and Lebanon. Mortality data among refugees in host countries were lacking. The available mortality data in Syria during conflict showed an increase in infant mortality and under-five mortality rates and maternal mortality ratio, although it was not possible to provide accurate data on conflict-related casualties among women, children and adolescents. Internationally, excess mortality due to conflict has been shown to be difficult to quantify,^49^ and the numbers are typically both difficult to obtain and disputed for political reasons.^50 51^

In Syria, available data show a decline in coverage of ANC, skilled birth attendance and child immunisation. Finally, as compared with rates in preconflict Syria, nutrition data demonstrate a decrease in undernutrition levels across all populations studied and an improvement in key IYCF behaviours in some populations in Syria and Jordan (although a decline in Lebanon).

A partial explanation for these observed trends may be differential access to healthcare across the study settings. IDPs in Syria have generally received less international attention compared with Syrian refugees. As noted above, none of the published articles identified through the literature search reported on the health of the internally displaced within Syria. Two-thirds of primary healthcare centres^14^ and 43% of public hospitals in Syria were reported to be fully functioning, with others either destroyed or compromised by severe shortages and overburdening of staff and medical equipment.^52^ Syrian refugees residing in host countries where health resources were already strained have difficulties accessing key services, largely due to cost and lack of knowledge or confusion about health services for refugees.^31 53–55^ Box 1 highlights differential access and entitlement to healthcare services in the different countries hosting Syrian refugees.

Box 1 **Differential access to healthcare in countries hosting Syrian refugees**

Although Syrians in Lebanon have the same healthcare access rights as Lebanese citizens,^80^ a recent survey of healthcare access among Syrian refugees (2015) found that only 24%–36% perceived healthcare as affordable and accessible, with 96% citing cost as the primary reason for not seeking healthcare for their children.^53^ The privatised, largely sectarian and comparatively expensive healthcare system is a primary barrier for care seeking.^31^ In Turkey, camp-based refugees can access healthcare through field hospitals and clinics.^81^ Non-camp refugees can access medical treatment from public hospitals, mother and child centres, and public or NGO-run clinics largely free of charge, and can obtain pharmaceuticals at 20% cost.^55^ Despite this, only about 60% of non-camp Syrian refugees accessed health services in Turkey, compared with over 90% of camp-based refugees.^41^ Language was a major barrier in accessing and providing healthcare in Turkey, with implications for refugees’ knowledge of existing health services and on vaccination coverage.^82^ The Turkish government has recently allowed Syrian doctors to staff its newly established migrant health clinics to redress linguistic barriers.^83^ In Jordan, camp-based refugees can access healthcare through UN and NGO-supported health services free of charge, whereas non-camp refugees (83% of Syrian refugees) access healthcare through a mix of government and private facilities.^84^ In camps, there is a referral system in place to transfer patients to hospitals outside the camp when medical services are lacking.^64^ Although healthcare was provided free of charge for all non-camp Syrian refugees in Jordan through 2014, it became not fully subsidised in 2015, resulting in reductions in access from 96% to 87% between 2014 and 2015.^54^ Lack of refugee registration, with UNHCR or AFAD, reduces access, particularly in Turkey where 31% of non-camp refugees are unregistered and an identification number is required to use hospitals.^41^

Most critically, our comprehensive review has found that in conflict-affected populations, coverage rates of key RMNCH interventions and health indicators are often unknown or difficult to ascertain. As we have shown, the vast majority of literature on these concerns has been focused on the Syrian refugees, often for reasons of access, and only a minority of the published literature is data driven. Big gaps remain in knowledge on public health within Syria during the conflict. A lack of representative primary data, inconsistencies across sources and measurement challenges were identified in addition to an inability to determine relevant denominators in such a dynamic setting. Where trend data were available, it was difficult to assess whether observed changes were real or a product of the changing nature of denominators.

Among the 12 articles identified by this review that included relevant primary or secondary data, only four articles had their first author based in a host country of refugees. It is particularly concerning that an academic public health team based in a conflict setting—Syria and its neighbouring countries—was not able to obtain data that were known to be available but were inaccessible despite extensive efforts by email and in person. Collaborations need to be encouraged across humanitarian agencies, governmental bodies as well as academic institutions in the regions affected by conflict in recognition of the potential comparative advantage and contributions of each type of institution.

This case study on the RMNCH needs of middle-income conflict-affected countries, where a minimum standard of care exists, shows that health challenges differ substantially from those faced by lower income countries with weaker infrastructure; IDPs and refugees from the Syrian conflict are not the typical camp dwellers seen in other conflict settings, but rather are often dispersed and integrated into host communities (with whom they share a common language in the case of Lebanon and Jordan), predominantly in urban areas. This necessitates creative approaches to reaching and addressing the health needs of these populations. At the same time, the nature of host-country health systems influences both entitlements of displaced populations and data collection on their health conditions given differences in national health information systems.

At the global level, our analysis identifies many challenges in systematically extracting data on key internationally agreed health indicators of the forcibly displaced. The fact that many institutions active in humanitarian contexts do not always use standardised, evidence-based definitions (in this case, the Countdown to 2015^6^ definitions of indicators) and do not always specify their methodology, including the sampling approach, has hampered our ability to draw valid conclusions.

A great deal of resources is currently being spent on data assessing health needs among Syrian refugees and the IDPs, yet this research could be more useful to inform public health interventions if it were more widely shared, if methodologies were more explicit and if comparable definitions and indicators were used and denominators clearly stated. The reluctance to share data across humanitarian agencies, governmental bodies and academic institutions must be overcome urgently for more effective responses, although there are some efforts underway to develop data-sharing systems in humanitarian contexts.^i^

Who is accountable for the health and well-being of the forcibly displaced? And who is responsible for collecting and reporting data on these populations? Within Syria, public health responsibility is deeply fragmented between government and opposing forces, which run parallel health systems with shifting geographic areas of coverage, and with available data often being restricted to government-controlled areas. Were full data disclosure and availability possible in this conflict context, with data-sharing across opposing forces and between countries, the results obtained here could well be worse, but at present this cannot be determined. In neighbouring countries, as in most complex emergencies, there remain ambiguities around accountability and problems of coordination between multiple humanitarian agencies and host governments. At the same time, the impact of the political conflict and influx of refugees undermines administrative capacity and reach of government services in host countries. Moreover, their accountability to the nationals of their country—and the competing needs of ‘host communities’ who are often the poorest and least well served—creates reluctance to underscore the needs of the refugees. Both governments and humanitarian agencies lack incentives to adopt more inclusive approaches that would seek input from the displaced themselves to inform service delivery or support community organisations to address their own needs. Legal restrictions on employment especially limit the ability of Syrian professionals and others within refugee communities to respond to community needs. There is evidence, however, that this trend is changing; Turkey has recently granted work permits to Syrian refugees^56^ and has allowed Syrian doctors to staff its newly established migrant health clinics. Jordan is also under international pressure to formalise employment for Syrian refugees.^57^

It is critical to include the forcibly displaced in global health accountability frameworks. The SDGs, as presently formulated, do not accord enough importance to the forcibly displaced, a topic that is covered in the preamble of the final SDG document but is not a subject of the final goals.^3^ Moreover, as in the earlier MDGs, the monitoring process foreseen for the SDGs as yet makes no distinction between citizens and non-citizens in data collection or in goals (even in countries with a very high proportion of non-nationals).^3^ States have clear obligations to produce vital statistics on their entire resident population, differentiated by nationality.^58^

As noted at the outset, Syria did not fit data-based inclusion criteria for the Countdown to 2015^6^ initiative, although were national data to become available, it could be eligible for inclusion today. Syria’s experience points to the fact that conflict may undermine, and even reverse, past progress towards achieving global RMNCH goals yet information on conflict settings is often lacking. In this paper, the Countdown to 2015^6^ framework of indicators was used as a lens to analyse the devastating effects of the Syrian conflict on the health of women and children because this framework represents internationally agreed indicators on which countries are required to report for global monitoring purposes. Although Countdown indicators are most useful as development indicators for low-income contexts where RMNCH is poor, there remains a need to ensure that a set of agreed RMNCH indicators are included when data are being collected in humanitarian contexts.

## Footnotes

i Such as the Global Humanitarian Data Exchange (https://data.hdx.rwlabs.org/) and the Syrian Regional Refugee Response—Inter-agency Information Sharing Portal (http://data.unhcr.org/syrianrefugees/regional.php).
