# Supplementary material for: Reproductive, maternal, neonatal and child health in conflict: a case study on Syria using Countdown indicators
Source: BMJ Glob Health. 2017 Sep 14;2(3):e000302. doi: 10.1136/bmjgh-2017-000302 (PMC5717942; doi:10.1136/bmjgh-2017-000302)
Supplement: Supplementary file 1 [file bmjgh-2017-000302supp001.pdf]

Appendices:

Appendix 1: Medline search strategy

Searches	Results	Search Type
1	premature birth.mp. or Premature Birth/	8463
2	gender based violence.mp. or Domestic Violence/ or violence against women.mp. or GBV.mp. or VAW.mp.	6852
3	(under-five or 0-59 months or 0- 60 months or <5 or less than five or less than 5).mp.	2061368
4	mortality rate.mp. or Mortality/	86585
5	3 and 4	14832
6	child mortality.mp. or Child Mortality/	3858
7	Pregnancy in adolescence/ or (Adolescent/ and birth rate/) or teen pregnancy.mp. or adolescent fertility.mp.	8258
8	Fertility/ or fertility.mp. or fertility rate.mp.	73725
9	stillbirth.mp. or Stillbirth/	5749
10	Infant Mortality/ or infant mortality.mp. or neonatal mortality.mp. or newborn mortality.mp. or NNM.mp. [mp=title, abstract, original title, name of substance word, subject heading word, keyword heading word, protocol supplementary concept word, rare disease supplementary concept word, unique identifier]	30248
11	under five mortality.mp.	253
12	5 or 6 or 11	18319
13	birth rate/	8022
14	birth certificates/ or birth registration.mp.	1402
15	1 or 2 or 7 or 8 or 9 or 10 or 12 or 13 or 14	144417
16	maternal death*.mp. or Maternal Death/ or Maternal Mortality/ [mp=title, abstract, original title, name of substance word, subject heading word, keyword heading word, protocol supplementary concept word, rare disease supplementary concept word, unique identifier]	10215
17	Maternal mortality/ or maternal mortality ratio.mp.	7956

18	16 or 17	10249
19	Prenatal Care/ or Perinatal Care/ or ((antenatal care or prenatal care or perinatal care) and (attendance or coverage)).mp. [mp=title, abstract, original title, name of substance word, subject heading word, keyword heading word, protocol supplementary concept word, rare disease supplementary concept word, unique identifier]	23367
20	((antenatal care or prenatal care or perinatal care) and (utili?ation or usage)).mp. [mp=title, abstract, original title, name of substance word, subject heading word, keyword heading word, protocol supplementary concept word, rare disease supplementary concept word, unique identifier]	1195
21	((antenatal care or prenatal care or perinatal care) and (access or visit*)).mp. [mp=title, abstract, original title, name of substance word, subject heading word, keyword heading word, protocol supplementary concept word, rare disease supplementary concept word, unique identifier]	3994
22	19 or 20 or 21	24580
23	Postnatal care.mp. or Postnatal Care/ or postpartum care.mp. or post delivery care.mp. or postpartum program*.mp. or puerperium care.mp. or postpartum cares.mp.	4791
24	18 or 22 or 23	37113
25	Delivery, Obstetric/	20873
26	obstetric deliver*.mp.	122
27	Obstetrics/ or obstetric.mp.	101066
28	emergency obstetric care.mp.	393
29	(skilled attendan* and (birth* or deliver*)).mp. [mp=title, abstract, original title, name of substance word, subject heading word, keyword heading word, protocol supplementary concept word, rare disease supplementary concept word, unique identifier]	195
30	25 or 26 or 27 or 28 or 29	101139
31	family planning.mp. or Family Planning Services/	43406
32	(family planning and (program* or use* or usage* or utili?ation or method*)).mp. [mp=title, abstract, original title, name of substance word, subject heading word, keyword heading word, protocol	33025

	supplementary concept word, rare disease supplementary concept word, unique identifier]	
33	31 or 32	43406
34	reproductive health services.mp. or Reproductive Health Services/	1834
35	access to essential services.mp.	19
36	Women/ or Child/ or Infant/	1648351
37	35 and 36	4
38	Cesarean section/	34767
39	(c?esarean section* or c?section* or abdominal deliver*).mp. [mp=title, abstract, original title, name of substance word, subject heading word, keyword heading word, protocol supplementary concept word, rare disease supplementary concept word, unique identifier]	47014
40	38 or 39	47014
41	abortion.mp. or Abortion, Induced/ or miscarriage.mp. or Abortion, Spontaneous/ or unwanted abortion.mp. or medical abortion.mp. or termination of pregnancy.mp. or self-induced abortion.mp.	74597
42	(PMTCT or Prevention of Mother-to-Child Transmission).mp. or Infectious Disease Transmission, Vertical/ or mother-child transmission.mp.	12422
43	22 or 24 or 30 or 33 or 34 or 37 or 40 or 41 or 42	270068
44	Tetanus Toxoid/ or Tetanus/ or Tetanus.mp.	24192
45	(neonatal or Newborn).mp. or Infant, Newborn/	659174
46	44 and 45	2585
47	Vaccination/ or Mass Vaccination/ or vaccination.mp. or Vaccination/ or Mass Vaccination/ or vaccination.mp. or vaccinate*.mp. [mp=title, abstract, original title, name of substance word, subject heading word, keyword heading word, protocol supplementary concept word, rare disease supplementary concept word, unique identifier]	120086
48	measles.mp. or Measles-Mumps-Rubella Vaccine/ or Measles/ or Measles Vaccine/	22218

49	47 and 48	7091
50	Tetanus Toxoid/ or Diphtheria-Tetanus Vaccine.mp. or Diphtheria Toxoid/ or Diphtheria-Tetanus Vaccine/	7881
51	Tetanus/ or Diphtheria/ or diphtheria.mp. or pertussis.mp. or Whooping Cough/	45274
52	47 and 51	7422
53	50 or 52	13472
54	PRP vaccine.mp.	48
55	Hib.mp. or Haemophilus influenzae type b/	2820
56	47 and 55	1315
57	54 or 56	1354
58	treatment for pneumonia.mp.	659
59	pneumonia.mp. or pneumonia/	107270
60	((lung or respirat*) and infect*).mp.	141985
61	58 or 59 or 60	224272
62	low birth?weight.mp. or Infant, Low Birth Weight/ or LBW.mp.	19014
63	wasting.mp. or Wasting Syndrome/ or underweight.mp. or stunting.mp. or wasted.mp. or stunted.mp. or under?nutrition.mp. or Malnutrition/ or malnutrition.mp. or malnourish*.mp. or acute malnutrition.mp. or chronic malnutrition.mp. or nutritional status.mp. or Nutritional Status/	82127
64	breastfeeding.mp. or Breast Feeding/ or breast?fe*.mp. [mp=title, abstract, original title, name of substance word, subject heading word, keyword heading word, protocol supplementary concept word, rare disease supplementary concept word, unique identifier]	30485
65	infant feeding.mp.	3543
66	(complementary feeding or complementary food*).mp. or Weaning/ or weaning.mp. or solid food*.mp. or infant food*.mp. or Infant Food/ or solid foods.mp.	37008
67	vitamin A supplementation.mp. or ((Vitamin A.mp. or Vitamin A/) and supplement*.mp.) [mp=title, abstract, original title, name of substance word, subject heading word, keyword heading word, protocol supplementary concept word, rare disease supplementary	4084

	concept word, unique identifier]	
68	49 or 52 or 53 or 57 or 61 or 62 or 63 or 64 or 65 or 66 or 67	400681
69	15 or 43 or 68	756983
70	Syria/ or syria.mp. or syria*.mp. or Syria.ti.	9690
71	69 and 70	387
**72**	**limit 71 to yr="2011 -Current"**	**50**

Table S.1: Status of Syrian refugees in host countries

	Lebanon	Jordan	Turkey
**Legal status of Syrian refugees**	-Lebanon did not ratify the 1951 Convention relating to the status of refugees and its 1967 Protocol, which removed geographical and temporal restrictions from the Convention. [1] Lebanon maintains the ability to refuse refugee status to foreigners coming to the country due to security reasons. According to the Ministry of Social Affairs (MOSA), a large number of Syrians (500,000) entered the country illegally since the start of the conflict.[2]	-Jordan did not sign the 1951 Convention relating to the status of refugees. Jordan has an open door policy for Syrian refugees irrespective of their entry status into the country.[1] The following groups of Syrians are not permitted to enter Jordan: Palestinians and Iraqis living in Syria, single men of military age and people who do not have legal documents.[1]	-Turkey signed the 1951 Convention relating to the status of refugees but had reservations related to "geographical limitations." Turkey does not grant non-Europeans the status of refugees. At the beginning of the crisis, refugees received a "temporary sheltering status". In March 2012, the government issued the first legal status about the status of Syrians "Directive on Receiving and Sheltering the Syrian Arab Republic Citizens and Stateless Persons Living in the Syrian Arab Republic Who Entered Turkey for the Purpose of Mass Sanctuary." The temporary shelter entails an open door policy, which does not force refugees to return to Syria. [2]
**Registration process and eligibility to assistance**	-Before registration with UNHCR was suspended by the government in May 2015[3], Syrian refugees were allowed to register with UNHCR whether they crossed an official border point or not. Refugees were required to bring any official document for each family member such as a national ID Card, a passport, etc. [1]<br><br>-Refugees registered with UNHCR receive basic health and social services.[4]	The UNHCR acts according to the 1998 Memorandum of Understanding with the Ministry of Interior in Jordan. UNHCR registration of Syrian refugees started after January 2012. UNHCR registered refugees have to register with the Jordanian authorities.<br><br>-Refugees registered with UNHCR receive an ID card that gives them access to cash and food aid.[2]	-Unlike Lebanon and Jordan where access to services depends on registration with UNHCR, access to services in Turkey depends on the Government.[1]
**Shelter accommodation and access to services**	- In January 2015, the total number of informal settlements reached 1470.[5] The settlements contain tents and makeshift houses.<br><br>-Unlike other neighbouring countries, the	In 2014, there were five camps/temporary centers for Syrian refugees. Three of them are permanent camps, and two are temporary settlements.[2] Camps have containers or tents and most have access to electricity and water.	-In 2014, there were 22 camps in 10 provinces in Turkey. [8] Camps have containers or tents.<br><br>-Camps are managed by the Turkish

	tented camps in Lebanon are not officially recognised by the government. Camps are organised by the local governments, which collaborate with NGOs to provide assistance to refugees. The living conditions in those camps are generally poor. [2]	-Camps are managed by UNHCR except for one camp. [2]	authorities, mainly by the Disaster and Emergency Management Presidency (AFAD), which is responsible for the infrastructure of the camps and their logistical facilities provision. AFAD is supported by other governmental institutions, which provide different services such as education, health and protection. [2]
	-Camps are much smaller than the camps in Jordan and Turkey, which were established by the respective governments because of the crisis.	-The living conditions of refugees living inside the camps vary according to the camp. For example, poor hygiene conditions were reported in Zaatari camp compared to the Emirates-Jordanian Camp, which is financed and managed by the United Arab Emirates.[6]	-Generally, the status of Syrian refugees living inside the camps is better than those living outside the camps. The camps are well organised, have better hygiene and better facilities than those in other countries hosting Syrian refugees.[2]
	-Sixteen percent of the Syrian refugees live in the settlements. The rest of the refugees live in households, often shared by many families, places that used to be shops or depots, or in buildings under construction. [2]	-Twenty percent of Syrian refugees live in the camps. The rest live in the cities with local communities.[7]	- In 2013. 36% of Syrian refugees lived inside the camps.[9] Refugees living outside the camps live in urban areas and vulnerable refugees live in collective shelters in mosques, wedding halls, and barns.[4]

References:

1. ACAPS. Legal status of individuals fleeing Syria - Syria Needs Analysis Project - June 2013.
2. ORSAM. Center for Middle Eastern Strategic Studies. The Situation of Syrian Refugees in Neighboring Countries: Findings, Conclusions and Recommendations2014.
3. UNHCR. Syria Regional Refugee Response Inter-agency Information Sharing Portal. 2015 [cited 2015]; Available from: http://data.unhcr.org/syrianrefugees/regional.php.
4. Bidinger S., Lang A., Hites D., et al. Protecting Syrian Refugees: Laws, Policies, and Global Responsibility Sharing: Boston School of Law.
5. The Relief Web. Syria Refugee Response Lebanon. Informal Settlements Location. 2015 [cited 2016 March]; Available from: http://reliefweb.int/sites/reliefweb.int/files/resources/UNHCR_LBN_SLT_MAP_2015-02-02_01_A0_InformalSettlementinLebanon.pdf.

6. World Health Organization Regional Office for the Europe. WHO Field Office in Gaziantep, Turkey. Monthly programme update - June 2016. Syria crisis2016.
7. UNHCR. 2015 UNHCR country operations profile - Jordan. 2015 [cited 2016 February]; Available from: http://www.unhcr.org/pages/49e486566.html
8. AFAD - Republic of Turkey Prime Ministry Disaster and Emergency Management Presidency. Syrian women in Turkey. 2014.
9. AFAD - Republic of Turkey Prime Ministry Disaster and Emergency Management Presidency. Syrian Refugees in Turkey, Field Survey Results2013.

Table S.2: Countdown policy indicators in Syria pre and post-conflict and in host countries post-conflict

	**Syria pre-conflict**	**Syria in conflict**	**Lebanon**	**Jordan**	**Turkey**
**International Code of Marketing of Breastmilk Substitutes**	Yes	Yes	Yes[1, 2]	Partial[3]	Yes
**New oral rehydration salts (ORS) formula and zinc for management of diarrhoea**	Partial	Partial	Partial	Partial	Partial
**Community treatment of pneumonia with antibiotics**	No	No	No	Data not obtainable	Yes
**Integrated management of childhood illness (IMCI) adapted to cover newborns ages 0–1 week**	No	No	No	No	Yes
**Costed implementation plan for maternal, newborn and child health available**	Partial	Partial	Yes	No	Yes
**Midwives authorised to administer a core set of life-saving interventions**	No	No	No	Yes	Yes

**Specific notification of maternal deaths**	No	No	Yes	Partial	Yes

Source: Key country informants unless specified otherwise.

References:

1. Akik C. Ghattas H, Filteau S, Knai C. Barriers to breastfeeding in Lebanon. Journal of Public Health Policy. 2017. doi:10.1057/s41271-017-0077-9
2. IBFAN Asia. The World Breastfeeding Trends Initiative (WBTi). Name of the Country: Lebanon2010.
3. World Health Organization. Country implementation of the international code of marketing of breast-milk substitutes: Status report 2011. 2013.

Table S.3: Financing indicators in Syria pre and post-conflict and post-conflict in host countries

	Syria pre-conflict	Syria in conflict	Lebanon	Jordan	Turkey
**Per capita expenditure on health ($)[1]**	97·0 (2011)	43·0 (2013)	631·0 (2013)	336·0 (2013)	608·0 (2013)
**General government expenditure on health as percentage of total government expenditure (%)[1]**	5·3 (2011)	5·3 (2013)	10·7 (2012)	13·5 (2013)	10·7 (2013)
**Out-of-pocket expenditure as percentage of total private expenditure on health (%)[2]**	100·0 (2010)	100·0 (2013)	69·5 (2013)	69·1 (2013)	66·3 (2013)
**Density of doctors, midwives and nurses per 10,000 population[1]**	Physicians: 14·6 (2010), Nurses and Midwives: 18·7 (2010)	9000 doctors have left the country as per the Syrian medical syndicate (Techreen 24/2/2014). This is on average 30% of the 30,000 doctors in the country.	Physicians: 32·0 (2011); Nurses and Midwives: 23·3 (2010)	Physicians: 25·6 (2010); Nurses: and midwives: 40·5 (2010)	Physicians: 17·1 (2011) Nurses and Midwives: 24·0 (2011)
**National availability of emergency obstetric care services**	6·7 per 500	N/A	N/A	N/O	N/O

N/A: not available (i.e. data do not exist to our knowledge)
N/O: not obtainable (i.e. data exist but authors unable to obtain)

References

1. Knoema. Global Health Observatory, 2015. 2015 [cited 2015]; Available from: http://knoema.com/WHOGHO2015/global-health-observatory-2015.

2. The World Bank. Out-of-pocket health expenditure (% of private expenditure on health). 2015 [cited 2015]; Available from: http://data.worldbank.org/indicator/SH.XPD.OOPC.ZS.
